# Face Recognition Deficits in Autism Spectrum Disorders Are Both Domain Specific and Process Specific

**DOI:** 10.1371/journal.pone.0074541

**Published:** 2013-09-11

**Authors:** Sarah Weigelt, Kami Koldewyn, Nancy Kanwisher

**Affiliations:** 1 Department of Brain and Cognitive Science and McGovern Institute for Brain Research, Massachusetts Institute of Technology, Cambridge, Massachusetts, United States of America; 2 Department of Psychology, Ruhr-Universität Bochum, Germany; University of Tuebingen Medical School, Germany

## Abstract

Although many studies have reported face identity recognition deficits in autism spectrum disorders (ASD), two fundamental question remains: 1) Is this deficit “process specific” for face memory in particular, or does it extend to perceptual discrimination of faces as well? And 2) Is the deficit “domain specific” for faces, or is it found more generally for other social or even nonsocial stimuli? The answers to these questions are important both for understanding the nature of autism and its developmental etiology, and for understanding the functional architecture of face processing in the typical brain. Here we show that children with ASD are impaired (compared to age and IQ-matched typical children) in face memory, but not face perception, demonstrating process specificity. Further, we find no deficit for either memory or perception of places or cars, indicating domain specificity. Importantly, we further showed deficits in both the perception and memory of bodies, suggesting that the relevant domain of deficit may be social rather than specifically facial. These results provide a more precise characterization of the cognitive phenotype of autism and further indicate a functional dissociation between face memory and face perception.

## Introduction

Dozens of studies [1] have found that face recognition is impaired in people with autism spectrum disorders (ASD). But what exactly is the nature and scope of this deficit? Is it “process specific”, that is, a problem only in remembering faces, or only in perceptually discriminating them? Second, is the deficit “domain specific”, that is, a problem recognizing faces per se, or does it extend to all social stimuli, or even more broadly to any category of visual object? The answer to these questions is important for two reasons. First, to understand autism itself we need a clear characterization of its cognitive phenotype/s, as well as a determination of which features of that phenotype constitute the causal core of the disorder, from which other features derive. Second, the fractionation of cognitive abilities in autism can reveal functional dissociations in the architecture of all minds. Thus, we tested the process specificity and domain specificity of the face recognition deficit in ASD by testing each of a relatively large sample of children with autism, and age and IQ-matched typical children, on both perception and memory of faces, cars, bodies, and places.

What does the existing literature say concerning the domain specificity of face recognition impairments in ASD [1]? In a recent review, we identified sixteen studies comparing face identity recognition (henceforth referred to as “face recognition) to the recognition of other visual objects in ASD, as needed to test the domain specificity of the deficit. Twelve of these studies report domain-selective deficits in which face recognition was more impaired than recognition of visual patterns [2]–[4], cars [5], [6], buildings [5]–[8], Greebles [9], common objects [9], shoes [10]–[12] and fans [13]. One additional study of 12 adults with ASD found that they performed worse than age-and IQ-matched adults with learning disabilities on memory for faces, but performed at least as well on memory for cats, horses, motorbikes, leaves and buildings [14] (That study also tested a group of adults that were age, but not IQ-matched to the group of adults with ASD. Because of the possible confound of IQ differences between groups, we do not consider the results of this control group). Although one very recent study found impairments for both faces and cars [15], as discussed further below, the literature overall suggests a selective deficit in ASD in recognition of faces compared to other categories.

However, because no study tested a non-face social stimulus, it remains possible that the deficit in ASD is not selective for faces per se, but extends to other social stimuli. Thus, in the present study we contrast the recognition of faces with a non-face social category: human bodies. Face and body recognition have been shown to be linked both in behavior [16], [17] and in the brain [18], making body recognition a particularly interesting contrast case to face recognition. We also tested two other non-social object categories: cars and places (or buildings). Both categories have been used in prior studies, which did not find recognition deficits for these categories in participants with ASD [5]–[18], but see [15] – thus we had a strong prior hypothesis that we would not find differences between ASD and TD groups in the recognition of cars or places.

What does the literature tell us on the question of process specificity of the face recognition deficit in ASD? Our recent review [1] found that deficits in face recognition in ASD are more often reported in studies with a high memory demand (face memory studies; [4], [7], [13]) than in studies with little or no memory demand (face perception studies; [19], [20]). One study used both a face memory and a face perception task on the same participants, and found that face recognition was more impaired when the faces had to be remembered over a delay than when they did not [10]. However these effects could reflect a greater across-the-board deficit in visual memory than visual perception in ASD, rather than a specific deficit in face memory.

Two prior studies tested both the domain specificity and the process specificity of face recognition deficits in ASD: Hauck and coworkers explored perceptual discrimination and memory for faces and houses in children with and without ASD and found significant interactions between group (ASD, TD), category (face, house), and group and task (memory, perception) [8]. Children with ASD performed worse on faces than houses and worse in the memory task than the perception task. Ewing, Pellicano and Rhodes compared perceptual discrimination and memory for faces (upright and inverted) and cars in children and adolescents with and without ASD [15]: They did not find interactions between group (ASD, TD) and category (face, inverted face, car), either for perceptual discrimination or for memory: Children with ASD performed worse than their age- and IQ-matched peers, both on faces and cars, and in perception as well as memory. Thus, the two studies show inconsistent results with regard to domain specificity and process specificity in ASD.

In the present study we aimed to resolve both questions by testing both the domain specificity and the process specificity of face recognition deficits in ASD. First, we crossed task (memory versus perception) with visual category (faces versus cars, bodies and places), in a 2×4 within-subjects design. Second, we used side views of cars, rather than front views, to avoid any visual resemblance to faces. Third, we tested a relatively large number of children (50 with ASD and 50 typically-developing children matched in age and IQ), to maximize our power to detect any deficits despite the notorious heterogeneity of autism. Fourth, we used the same stimuli for the memory and perception tasks, so that any differential deficits in ASD for memory versus perception can be attributed to the memory load, not to the stimuli. Finally, we used stimuli and tasks that have been optimized in a prior developmental study [21] to minimize floor and ceiling effects, thus providing maximum sensitivity to any differences between those with ASD and those without. Based on our recent review of the literature [1] we predict significant deficits in face memory in autism that would be greater than any deficits in face perception.

## Methods

### Participants

Participants were 50 typically developing children and 50 children with ASD aged 5–12 years (6 girls and 44 boys in each group). We matched the groups on age and non-verbal IQ, measured by the Kaufman Abbreviated Intelligence Test (see Table 1). Participants with ASD had an ASD diagnosis from a clinician and met criteria for ASD or autism on the Autism Diagnostic Observation Schedule (ADOS). A higher percentage of the ASD children were Caucasian (49 out of 50) than the typical control children (38 Caucasian, 11 Non-Caucasian, 1 unknown), so any other race effects [22] would go against the hypothesis of face deficits in ASD. All participants received modest monetary compensation for their participation as well as small motivating prizes. Children with ASD were recruited through the Simons Foundation and the Boston Autism Consortium. Typically developing children were recruited from the local community. Potential participants were excluded if they had any history of birth or brain trauma, non-corrected visual impairments or a non-verbal IQ of less than 80. Typically developing participants were further excluded if they had a diagnosis of any developmental disorder or any history of ASD in their immediate family.

**Table 1:** Participant information.

	Autism (n = 50)			Control (n = 50)			t	p-value
Measure	Mean	SD	Range	Mean	SD	Range		
Performance IQ (K-bit)	107.96	15.82	80–139	112.41	12.39	84–140	1.555	.123
Age	9.25	1.81	5.79–12.92	9.07	1.94	5.44–12.55	−0.495	.622
ADOS (severity score)	7.08	1.66	3–10	–	–	–	–	–

* Note that we were unable to obtain the IQ score of one TD child and the ADOS score of one ASD child.

### Ethics Statement

The study was approved by MIT’s Committee on the Use of Humans as Experimental Subjects and was conducted in accordance to the principles laid down in the Declaration of Helsinki. Every participant signed an assent form and a parent or guardian signed an informed consent.

### Standardized Measures

Data from these following standardized measures are presented in Table 1.

*Autism Diagnostic Observation Schedule (ADOS*) [23]: A structured observational assessment that provides opportunities for interaction and play while measuring social, communicative and repetitive behaviors that are diagnostic of ASD.

*The Kaufman Brief Intelligence Test (k-bit)* [24]: The K-bit provides a short and reliable means of assessing intelligence in individuals aged 4–90. Only the nonverbal subtest was used, testing skills such as pattern recognition, analogy completion and mental rotation.

### Design

The experimental design was the same as a prior study conducted on typical children [21]. Each participant was tested in each of two tasks (memory and perceptual discrimination) on four stimulus categories (faces, cars, bodies, and places). Memory was always tested before perception so that exposure to the stimuli in the perception task would not affect memory performance for those stimuli.

### Procedure

Stimuli were shown on a MacBook Pro or Elo touchscreen computer using Matlab and the Psychtoolbox extension (version 3.0.9, [25], [26]). Participant responses were recorded either via touchscreen or buttons. Some children gave their answers by pointing towards the screen, and the experimenter pressed the buttons accordingly. Because these different response devices were used, we use only accuracy, not reaction time, as a dependent measure. Participants were tested individually.

### The Memory Task

The memory task was conducted in two segments.

#### Segment 1: faces and cars

**Figure 1:**
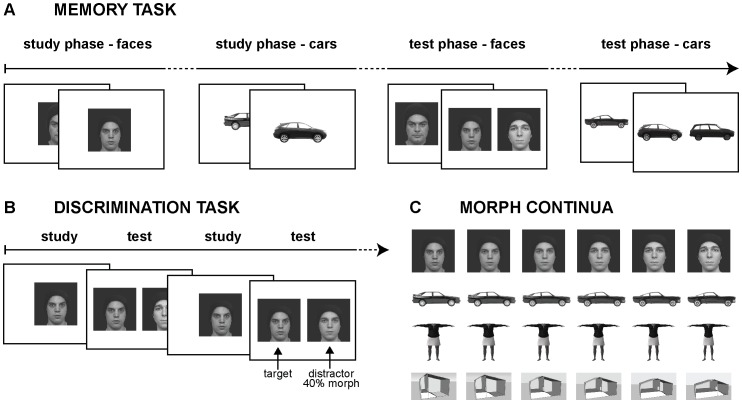
Experimental paradigms. A) The memory paradigm. A study phase consisting of 10 items per category (faces and cars, or bodies and places), each presented for 3 s at the center of the screen, was followed by a 2AFC test phase consisting of 10 pairs of stimuli per category (one studied, one new). B) The perception paradigm. Perceptual discrimination was tested for the four categories (faces, cars, bodies, and places) via an immediate 2AFC match-to-sample task. In detail, a target item was presented for 1 s at the center of the screen, and immediately followed by a test pair of stimuli side-by-side. Each test pair included the target item and a distractor item created by morphing the target item towards a different-identity exemplar of the same category. C) Morph continua. One example from each stimulus category for the morph continua is shown. There were ten morph continua per category.

In the first segment, participants studied ten face items followed by ten car items. During the study phase participants were told to observe all items carefully and to remember them as best as they could. The experiment started with a fixation cross which was displayed until the experimenter judged the participant was concentrating. Then the twenty study items were presented sequentially at screen center for 3 s each. Following the study phase was a 2AFC test phase (Figure 1A), with 10 face pairs followed by 10 car pairs. Items in each test pair were shown simultaneously side-by-side until the participant responded which one was “old” (seen previously), guessing if necessary. Each trial consisted of one old and one new/distractor item. The old item was on the right 50% of the time. Items in the test phase appeared in the same order as in the study phase to approximately equate memory delay across items (about 1 minute between study and test). No feedback was provided. The dependent measure was accuracy. Chance is 50%.

#### Segment 2: Bodies and places

The procedure of the first segment was repeated for the second segment testing bodies and places (bodies were first for both study and test).

### The Perceptual Discrimination Task

We measured perceptual discrimination threshold (the stimulus difference necessary to perform 75% correct) in a 2AFC match-to-sample task (Figure 1B). Each participant was tested on all four stimulus categories, in the order faces, cars, bodies places. On each trial, a fixation cross was presented until the experimenter judged the participant was concentrating. Then the study item appeared at screen center for 1 s, followed by a test pair presented simultaneously side-by-side until the participant responded. The test pair comprised the study item and a distractor created by morphing the target item towards a different-identity exemplar of the same category (Figure 1C). The different-identity exemplar was never a target item, but only a distractor exemplar to this one target item. Participants were told to report the item they had just seen.

#### Experimental trials

The dependent measure was the morph distance at which participants could discriminate between the study and test items with an accuracy of 75% correct. This was estimated using a QUEST staircase [27] with parameters: number of trials = 30; Beta (slope of the estimated psychometric function) = 3.5; Delta (estimated probability of a failure well above threshold) = 0.01; Gamma (estimated probability of a correct response at zero intensity) = 0.5; Grain (intensity steps, i.e. minimum morph difference between two images) = 5. Participants were told the task would get harder as they went along, until they might not be able to tell the difference between the two items, at which point they should guess. Children were told not to feel bad about not knowing which is the correct one. The correct item was on the right 50% of the time. No feedback was provided.

#### Practice trials

At the beginning of each category we used very easy test pairs (the target item and an 80%-morph distracter) for practice. Child participants received feedback and encouragement. If children got four in a row correct, the program advanced to the experimental trials. If a child was unable to complete any four consecutive trials of 12 trials total, a new practice session started; if unable to complete any of three practice sessions, this was counted as the child not being able to perform the discrimination task (for that particular category – the other categories were still tried and tested). This occurred in ten participants (TD children: two for bodies, one for places; ASD children: one for places, four for bodies, one for faces and one for faces and bodies). The respective data points were replaced with the lowest score measured across any age or participant group. The results do not change qualitatively when these children are excluded from the analysis.

#### Lapse trials

Six lapse trials per category were interspersed at regular intervals among the experimental trials. These contained very easy test pairs (study item and a 100%-morph distracter) and were included to make sure children understood and continued to perform the task.

### Stimuli

All stimuli were grayscale, static and not displaying emotion (relevant to faces and bodies), displayed in ‘canonical’ viewpoint, and always presented in the same images at study and test. Faces were natural, real world faces of Caucasian men from the Harvard Face Database, with neutral expression, in front view, sized 11.137° visual angle vertical×11.137° horizontal including the black background, with no facial hair or glasses, and black hats to hide hair and ears. The persons shown in Figure 1 have given written informed consent, as outlined in the PLOS consent form, to publication of their photographs. Cars were photographs of typical cars, in side view, sized 7.153° vertical×16.63° horizontal, on a white background. Car stimuli were freely available images from the Internet and were chosen to allow morphing between relatively similar pairs. The headless bodies were images of human adults (50% female) generated with Poser 6 software (Smith Micro Software Inc., Watsonville, CA, USA), in front view, in constant pose (i.e., arms out), not expressing emotions, on white background. The size of the bodies varied between 13.544° and 15.658° vertically, 4.295° and 7.153° horizontally with respect to the torso width and 17.061° and 19.852° with respect to the arm width. Places were perspective views of houses missing one wall, sized 9.25° vertical×14.955° horizontal including a shaded background, and generated with Google SketchUp.

The 20 individual stimuli of each category were paired to make 10 morph continua, by morphing one endpoint exemplar into its paired exemplar (e.g. one face into its paired face, see Figure 1C) in steps of 5%. Morphing was realized within FantaMorph Software (Abrosoft) for faces and cars, Poser 6 for bodies (only between stimuli of the same gender with same clothing), and Google SketchUp for places.

## Results

### Face and Body Memory Deficits in ASD Children

**Figure 2:**
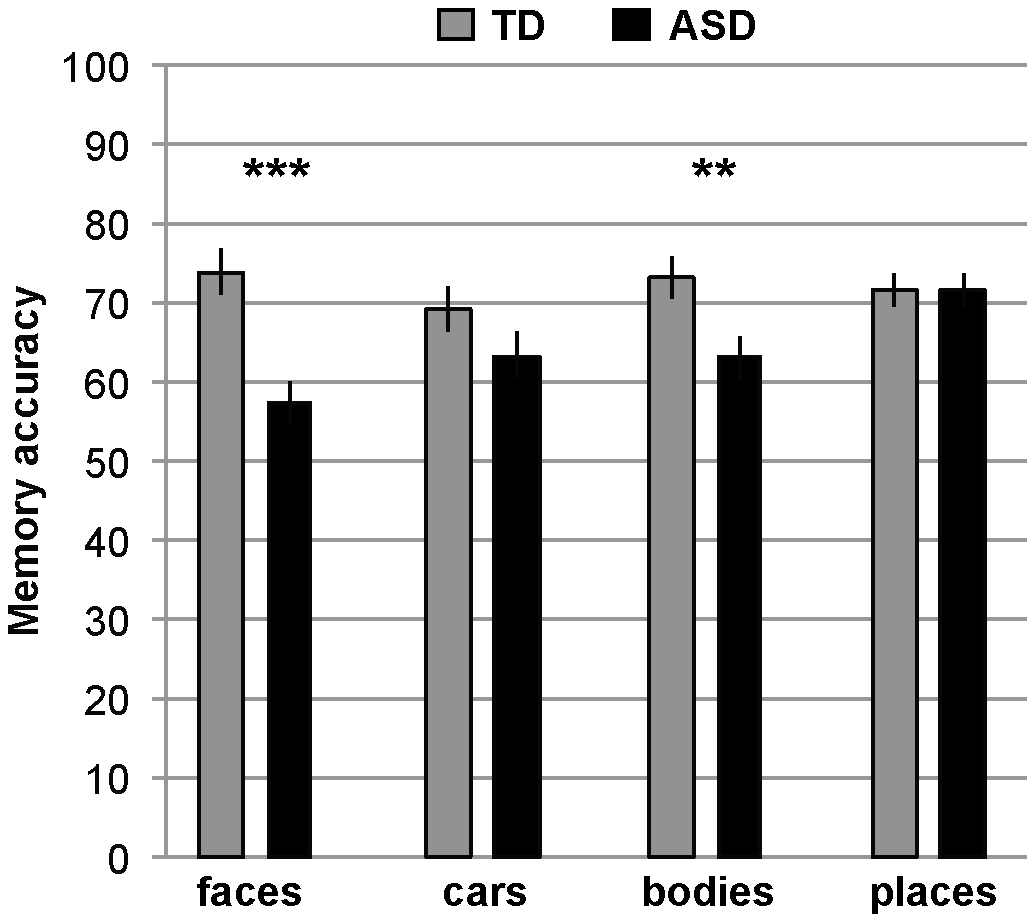
The memory results. Memory accuracy in percent correct for each of the four stimulus categories for children with ASD versus age and IQ-matched typical children. Results indicate a deficit in face and body memory in children with ASD. Chance was 50% indicated by the horizontal line. Error bars denote SEM. ** denotes p<.01, *** denotes p<.001.

Results for memory (Figure 2) indicate deficits in face and body memory in children with ASD compared to TD children, but similar performance for car and scene memory. An ANOVA including group (ASD, TD) and within-group factor category (faces, cars, bodies, places) revealed a main effect group, *F*(1, 98) = 11.349, *p = *.001, η_p_^2^ = .104, indicating worse performance in children with ASD in comparison to the TD children, a main effect category, *F*(3, 294) = 2.645, *p = *.049, η_p_^2^ = .026, and crucially a significant group×category interaction, *F*(3, 294) = 5.802, *p = *.001, η_p_^2^ = .056. Follow-up independent sample T-Tests contrasted the performance for ASD and TD children for each other category in turn. Children with ASD performed significantly worse than TD children in face, *T*(98) = 4.640, *p* = .000, and body, *T*(98) = 2.714, *p* = .008, memory, but performed similarly in scene memory, *T*(98) = −0.064, *p* = .949. ASDs showed a nonsignificant trend of lower memory for cars, *T*(98) = 1.665, *p* = .099, but importantly the deficit for faces was significantly greater than for cars, as demonstrated by a significant interaction of group (ASD, TD)×category (faces, cars), *F*(1, 98) = 5.229, *p* = .024, η_p_^2^ = .051).

To ensure that IQ differences were not influencing our results, we re-ran the ANOVA with IQ as a covariate. The group (ASD, TD)×category (faces, cars, bodies, places) interaction remained significant, *F*(3, 288) = 4.742, *p = *.003, η_p_^2^ = .047.

Reinforcing our hypothesis that memory deficits in autism selectively affect social compared to nonsocial stimuli, the interaction of group (ASD, TD)×social (faces, bodies) versus nonsocial (cars, places) was also significant, *F*(1,98) = 11.040, *p* = .001, η_p_^2^ = .101).

### Body Perception Deficits in ASD Children

**Figure 3:**
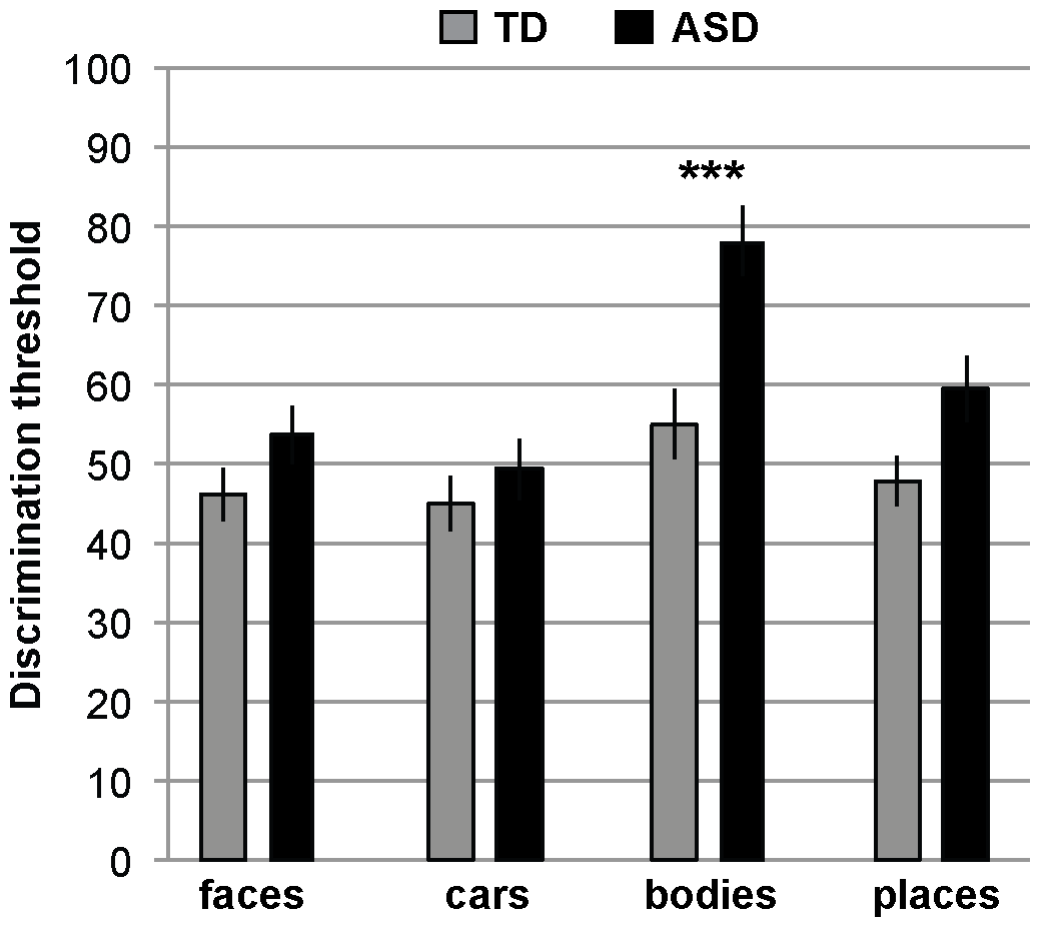
The perception results. Perceptual discrimination threshold (morph-level difference necessary to perform at 75% correct) for the four categories for children with ASD versus age and IQ-matched typical children. Results indicate deficits in body perception in children with ASD. Error bars denote SEM. *** denotes p<.001.

Results for perception (Figure 3) indicate deficits in body recognition in children with ASD in comparison to TD children, but similar performance for face and car perception, and a trend toward a deficit for scene perception. Note that higher scores signal worse performance. An ANOVA including group (ASD, TD) and within-group factor category (faces, cars, bodies, places) revealed a main effect group, *F*(1, 98) = 8.474, *p = *.004, η_p_^2^ = .080, indicating worse performance in children with ASD in comparison to the TD children, a main effect category, *F*(3, 294) = 12.664, *p = *.000, η_p_^2^ = .114, and, importantly, a significant group×category interaction, *F*(3, 294) = 2.731, *p = *.044, η_p_^2^ = .027. Follow-up independent sample T-Tests contrasted the performance for ASD and TD children for each other category in turn. Children with ASD performed significantly worse than TD children in body perception, *T*(98) = −3.566, *p* = .001. Children with ASD performed similar to TD children on face, *T*(98) = −1.371, *p* = .174, car, *T*(98) = −0.768, *p* = .444, and scene, *T*(98) = −1.518, *p = *.132 perception. Note that the null-effect for face perception is unlikely to represent a deficit in face perception masked by an other-race-effect in the opposite direction (given the higher percentage of Caucasian participants in the typical group and the use of Caucasian faces as stimuli): In a comparison between groups matched for race (in a subset of 32 Caucasian children with ASD and 32 Caucasian typical children), children with ASD still performed similarly to typical children, *T*(62) = −0.782, *p* = .437.

To ensure that IQ differences were not influencing our results, we re-ran the ANOVA with IQ as a covariate. The group×category interaction remained significant, *F*(3, 288) = 2.749, *p = *.043, η_p_^2^ = .028.

An ANOVA with group (ASD, TD)×social (faces, bodies) versus nonsocial (cars, places) revealed a non-significant interaction, *F*(1,98) = 2.490, *p* = .118, η_p_^2^ −.025) supporting the conclusion that the deficits in perception are mainly driven by one category (bodies) rather than the ‘socialness’.

### Direct Comparison of Memory and Perception Performance

The analyses reported above indicate a significant deficit for face memory but not for face perception in children with ASD, but these statistics are not sufficient to demonstrate that face memory is *significantly more* impaired than face perception [28]. To directly compare performance between the memory and perception tasks we standardized each ASD child’s performance by expressing it in units of standard deviation from the mean of all typical children’s performance. That is, we standardized each ASD child’s memory performance by subtracting the mean performance of the TD children and dividing the result by the standard deviation of the TD children, Standardized_score_ASD_ = (Original_score_ASD_ – Mean_TD_)/SD_TD_. Correspondingly, we standardized each ASD child’s perception performance by subtracting the mean performance of the TD children from the original score and dividing the result by the standard deviation of the TD children, Standardized_score_ASD_ = (Mean_TD_ - Original_score_ASD_)/SD_TD_. Because this procedure put scores for both memory accuracy and perceptual threshold in the same units of SDs from the typical population, we could conduct direct statistical comparisons of deficits in memory and perception.

**Figure 4:**
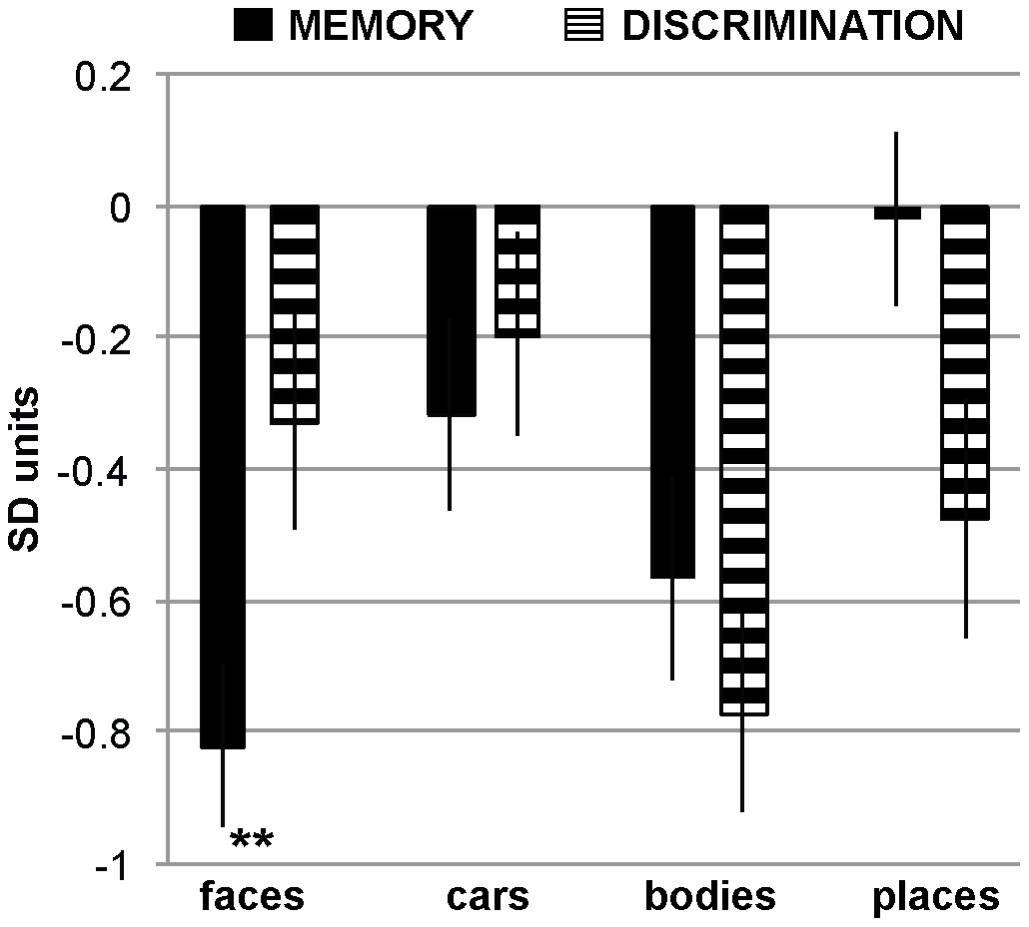
Direct comparison between memory and perception data. Mean across ASD participants of the standardized score for each participant (i.e., the standard deviation of each participant from the typical population) for each stimulus category for memory (left) and perception (right). Values below zero indicate an impairment in ASD. ** denotes p<.01.

Results are depicted in Figure 4. Negative SD units indicate worse performance in ASD compared to the TD group. An ANOVA with the within-group factors task (memory, perception) and category (faces, cars, bodies, places) revealed a main effect category, *F*(3, 147) = 5.754, *p = *.001, η_p_^2^ = .105, but no main effect task, *F*(1, 49) = 0.421, *p = *.520, η_p_^2^ = .009, and crucially a significant task×category interaction, *F*(3, 147) = 5.724, *p = *.001, η_p_^2^ = .105. Follow-up independent sample T-Tests contrasted the performance between tasks for each category in turn. As predicted, children with ASD performed significantly worse in face memory than in face perception, *T*(98) = −2.862, *p* = .005. We did not observe differences between memory and perception for cars, *T*(98) = −0.833, *p* = .407, bodies, *T*(98) = 0.747, *p* = .457, or places, *T*(98) = 1.620, *p* = .108. Thus, only for faces did we find a significantly greater impairment in ASD for memory than perception.

### Face Memory Deficits Correlate with Autism Severity

To assess potential relationships between recognition performance in our tasks and autism severity, we ran partial correlations (partialing out age and IQ) with our face, car, body, and scene measures of perception and memory (8 measures) and the ADOS severity score (ADOS CSS; [29]). Face memory performance showed a trend of a negative correlation with autism severity, r = −.277, df = 45, *p* = .060 indicating better face memory performance in less severely affected children. Puzzlingly, body perception threshold was negatively correlated with autism severity, r = −.298, df = 45, *p* = .042, indicating better body discrimination performance in more severely affected children, and raising questions about the interpretation of the perceptual deficit for bodies in ASD. All other *ps* are >.125.

### Summary

Our strongest result is a face recognition deficit in ASD that is specific for memory, not perception, and shows a borderline-significant trend of a correlation with autism severity. We also find body deficits in children with ASD, both in memory and in perception, although neither is correlated positively with ADOS severity.

## Discussion

Our results demonstrate that face recognition deficits in ASD are both domain-specific and process-specific. Domain specificity is demonstrated by a significant deficit in face memory for ASDs compared to TDs, in the absence of any parallel deficit for car or scene memory. Both perception and memory of bodies was impaired however, suggesting that the relevant domain may extend beyond faces to include other social stimuli. The process-specificity of the deficit is demonstrated by the fact that ASDs show no significant impairment in face perception, only in face memory. These findings, discussed in more detail below, more precisely characterize the cognitive phenotype of ASD and provide intriguing clues about the functional architecture of face processing more generally.

### Domain Specificity of the Face Deficit in ASD

Our results demonstrate that the face deficit in ASD is domain specific: accuracy was significant lower for face memory, but not for car or scene memory in comparison to TD individuals. This result agrees with most of the prior evidence [1]. In one notable exception, Ewing et al. recently reported deficits in both face memory and car memory in ASD [15]. One possible explanation of these conflicting results is that the car stimuli in the Ewing et al. study were front views of cars, which look somewhat like faces and might be processed by the face system. Future work could evaluate this hypothesis by testing whether inversion effects, which are not found for our side-view car stimuli (see ref. [21]), are found for the front-view car stimuli used by Ewing et al. [15].

In addition to their deficit in face memory, children with ASD were also significantly impaired in body memory, suggesting that the relevant domain is broader than faces, perhaps extending to all social stimuli. Another possibility is that our data reflect two distinct deficits, one for faces, and another for bodies. The “two deficits” hypothesis is consistent with the facts that i) the deficit for faces affects only memory whereas the deficit for bodies affects both memory and perception, and ii) the face memory deficit shows a borderline-significant trend of a correlation with ADOS severity but the body memory deficit does not, and iii) face memory performance is not correlated with body memory performance in ASD subjects (r<−0.09; *p*>0.5).

Only one prior study tested body identity perception in autism using static images, but that study only tested for body inversion effects [30], which have been shown to result from the face processing system [17], [31], [32]. However, deficits in body perception in ASD have been shown in dynamic “biological motion” stimuli (e.g. refs. [33]–[37]; though see ref. [38]). These stimuli depict body form and motion from only moving dots, but could reflect the same underlying deficit as that found here with static images. Perhaps these impairments in visual body representation are even linked to deficits in body coordination [39] and representation [40] in ASD.

### Process Specificity of the Face Deficit in ASD

Our finding that face impairments in ASD are process-specific for memory, not perception, accord with our recent review of the literature [1], which showed that face deficits are more robust in tests with high memory load (e.g. refs. [4], [7], [13]) than tests with low memory load. The three studies testing both memory and perception in the same subjects all found face memory to be more impaired than face perception [8], [10], [15], (One of these [15] reported impairments in both face memory and face perception, but the perceptual deficit in that study was small in effect size (approximately 82% correct in children with ASD versus 86% correct in TD children from their Figure 4A), statistically weak (*t*(78) = 1.93, p<.05, one tailed), and apparently smaller than the deficit in face memory (d’ of about.65 for children with ASDs versus 1.25 for TD children, *t*(78) = 3.56, *p*<.01).) Although small deficits in face perception may be found in studies with greater power than ours, our data accord with the literature in showing that face memory is more impaired in autism than is face perception. This dissociation between face perception and face memory has important implications both for ASD and for the functional architecture of the face system more generally, as discussed below.

### Implications for ASD

Our findings have several important implications for autism. First, the face memory impairment found here is particularly strong because it was hypothesized in advance based on our recent literature review [1], and because it shows a borderline-significant correlation with autism severity, strengthening the link to autism. Might an early-developing deficit in face memory play a causal role in the etiology of autism? Evidence against this idea comes from the finding that individuals with congenital prosopagnosia (whose face memory abilities are generally more impaired than those with ASD) are not impaired in social cognition [41]. Thus, face recognition impairments on their own are not sufficient to lead to autism. A more likely hypothesis is that face memory impairments are the result, not the cause, of the autism phenotype. For example, face memory deficits may result from underlying differences in social interest, or differences in eye movement strategies. The causal pathways underlying the etiology of autism may be best evaluated through longitudinal studies of infants and young children on face memory and other cognitive processes affected in ASD. It will also be of interest to find out whether face memory deficits in ASD persist into adulthood, or whether the deficits reported here reflect a developmental delay that is overcome later in life.

Second, our finding that both the perception and memory of body form are disrupted in autism is a new finding not reported previously. The relationship (if any) of this finding to face impairments and to impairments in the perception of biological motion remains to be explored.

Third, our data support the hypothesis that autism is fundamentally a domain-specific disorder that primarily affects social cognition (see also [42]). Although disorders of domain-general processes have been hypothesized to play an important and even causal role in autism, including deficits in global processing, dynamic attention, and attentional disengagement, evidence against widespread impairments in any of these processes has been accumulating in recent years [43]–[45]. It is unlikely that all aspects of the cognitive phenotype/s of autism will turn out to be social, but our findings support the hypothesis that deficits in social cognition lie at the core of autism.

### Implications for Functional Architecture

Beyond their implications for autism, our results provide clues about the functional architecture of high-level vision in general. Most importantly, the dissociation between face memory and face perception reported here suggests that these two phenomena rely on at least partly distinct mechanisms. Further evidence for this hypothesis comes from our earlier finding (using the same stimuli and tasks as in the present study) that face-specific memory develops between age 5 and adulthood in typical children whereas face-specific perception does not [21]. Further evidence for this dissociation comes from neuropsychology: In “prosopamnesia”, patients are impaired in face memory but not face perception, but not in either object memory or object perception [46], [47]. Finally, oxytocin administration specifically improves face memory, not memory for nonsocial stimuli [48], apparently by affecting face memory processes not face perceptual processes [49], echoing earlier studies showing that oxytocin plays a specific role in social memory in rodents [50]. The brain basis of the dissociation between face recognition and face memory is not known, but could reflect either the existence of a distinct brain region selectively engaged in face memory (perhaps in anterior temporal regions), or a disconnection between posterior temporal regions specific for face perception and medial temporal memory systems (e.g., a disruption of the inferior longitudinal fasciculus).

In sum, the present study elucidates the scope and nature of one of the most widely noted and replicated deficits in autism: face recognition. We find that this deficit primarily affects face memory, not face perception, and we further show that the deficit is specific to social stimuli. These results further underscore the well-established domain specificity of the machinery for face recognition in typical subjects [51]. Finally, the fractionation of the face system in autism (between perception and memory) provides an important clue into the functional architecture of face processing in typical brains.
